# Oncogenic MNK signalling regulates the metastasis suppressor NDRG1

**DOI:** 10.18632/oncotarget.17555

**Published:** 2017-05-02

**Authors:** Shuye Tian, Xuemin Wang, Christopher G. Proud

**Affiliations:** ^1^ Nutrition and Metabolism, South Australian Health and Medical Research Institute, Adelaide SA5000, Australia; ^2^ School of Biological Sciences, University of Adelaide, Adelaide SA5005, Australia

**Keywords:** NDRG1, MNK1, SGK, migration, breast cancer

## Abstract

The protein N-myc down-regulated gene 1 (NDRG1) represses tumour metastasis. It is phosphorylated at several sites by serum and glucocorticoid-regulated kinase 1 (SGK1). Here we show that NDRG1 is also regulated by the oncogenic MAP kinase-interacting kinase (MNK) pathway, a target for cancer therapy.

Inhibiting MNKs increases the expression of NDRG1 protein and mRNA in breast cancer cells. MNK inhibition also decreases the phosphorylation of NDRG1. Phosphorylation of NDRG1 is reduced in cells lacking MNK1, but not MNK2-knockout cells, indicating that NDRG1 phosphorylation is a specific target for MNK1. However, MNK1 cannot directly phosphorylate NDRG1 *in vitro*, indicating that additional signalling connections are involved. Taken together, our data indicate that MNK signaling regulates NDRG1 at transcriptional and post-translational levels.

We show that SGK1 phosphorylates MNK1 at a conserved site, which represses its activity. NDRG1, SGK1 and the MNKs are implicated in cell migration and metastasis. As expected, knocking-down NDRG1 promoted cell migration. However, whereas MNK inhibition impairs these processes irrespective of NDRG1 levels, SGK inhibition only did so in NDRG1-depleted cells. Thus, MNKs and SGK affect migration/invasion through distinct mechanisms.

Our data reveal several novel connections between signalling pathways important for tumour biology.

## INTRODUCTION

The protein N-myc downstream regulated gene 1 (*NDRG1*) is widely expressed in mammalian cells, especially epithelial cells. NDRG1 has been reported to be a tumour suppressor and/or negative regulator of metastasis in various cancers, including breast cancer [12]. NDRG1 is also involved in other processes related to oncogenesis and tumour metastasis (reviewed [3]). For example, it is a differentiation marker in breast cancer [4].

NDRG1 undergoes phosphorylation at several sites including well-conserved ones at Thr346, 356 and 366 by serum and glucocorticoid-regulated kinase 1 (SGK1) [2]. However, the role of NDRG1 phosphorylation remains unclear, as does its biological function [1, 5]. In particular, it is unclear whether NDRG1 phosphorylation affects its role as an inhibitor of migration.

Recent data show that the MAP kinase-interacting kinases (MNKs [6, 7, 8]) also regulate cell migration, but in this case, they promote migration [9], although it remains to be determined how they do this.

There are two MNK genes in mammals; in humans, each gene transcript can be alternatively spliced, giving rise to two proteins. The longer forms, MNK1a/2a which differ from the shorter ones (MNK1b/2b) at their C-termini [8] contain a MAP kinase binding site.

Human MNK1a and its equivalent in mice, MNK1, are tightly regulated by the upstream kinases ERK and p38 MAP kinase [8, 10, 11] whereas MNK2a/MNK2 have high basal activity [10].

Although MNKs were discovered almost twenty years ago [6, 7], there is so far only one *in vivo*-validated direct substrate for them, eukaryotic initiation factor (eIF) 4E, which they phosphorylate at Ser209 [7, 12, 13] Both MNK1 and MNK2 phosphorylate this substrate [14], and there are no proteins that are known to be controlled specifically by only one MNK. Other MNK substrates have been identified *in vitro* including some involved in gene expression or its control [15, 16]. Further substrates may exist.

There is strong interest in the MNKs’ role in tumorigenesis and tumor progression, as they are activated by oncogenic Ras/Raf/MAP kinase signalling [17]. Lack of the MNKs, or of eIF4E phosphorylation, delays development or progression of certain solid tumors (e.g., prostate cancer and glioma) [18, 19]. However, little is known about the relevant molecular mechanisms and downstream targets of the MNKs involved.

The MNKs have emerged as potential therapeutic targets in oncology and there are now substantial efforts to identify small molecule inhibitors of these enzymes [17, 20].

We were therefore interested to gain further insight into the roles of the MNKs in cancer cells, their interaction with other oncogenic signaling processes and the mechanisms by which they affect cell migration.

## RESULTS

### MNK inhibition regulates the overall expression and phosphorylation of NDRG1 in MDA-MB-231 breast cancer cells

Our earlier data showed that MNKs promote cell migration [9]. However, it is unclear how they do this. We therefore asked whether MNKs regulate NDRG1, a phosphoprotein that negatively affects cell migration and tumour metastasis [5].

**Figure 1:**
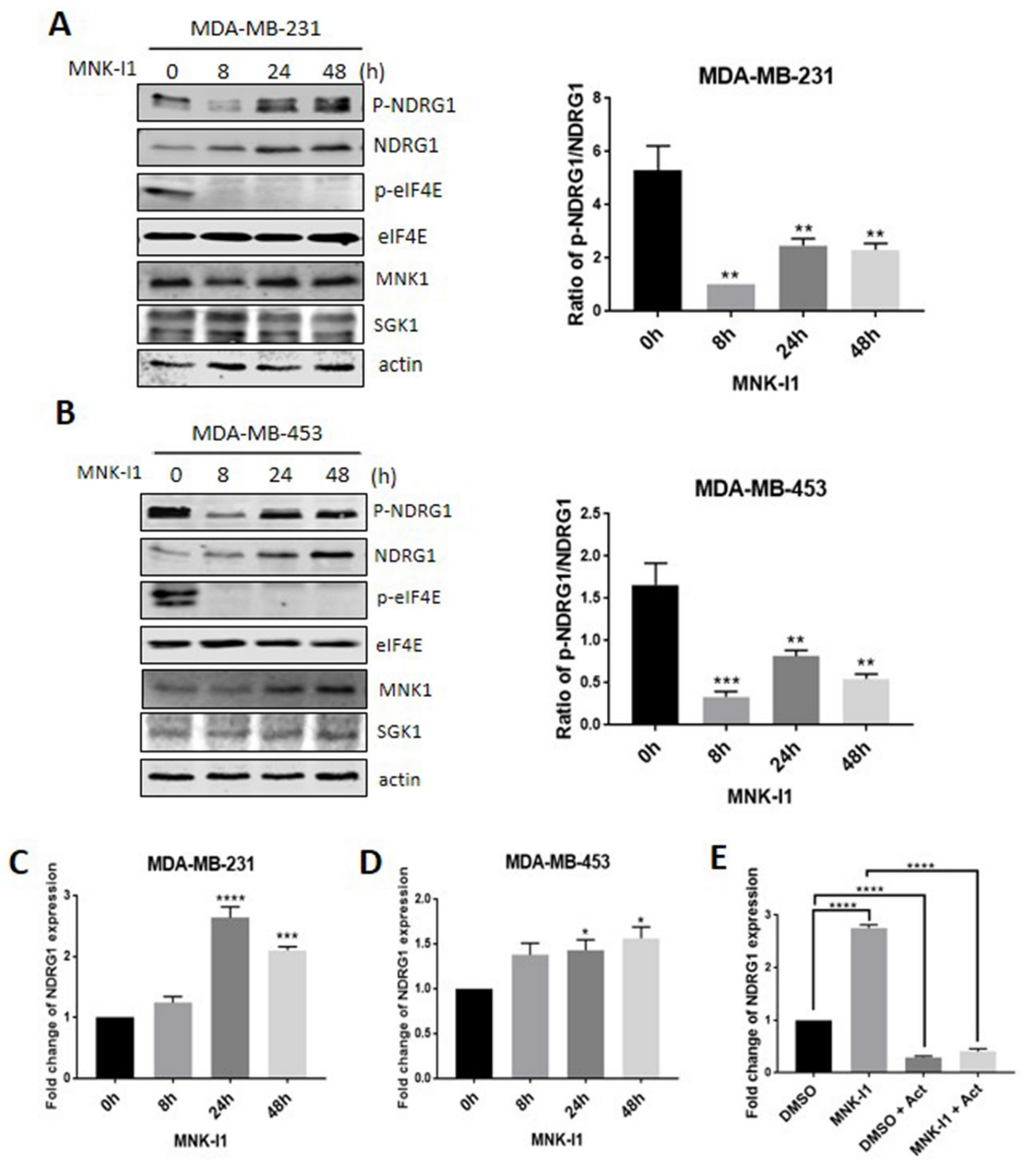
MNK inhibition regulates the phosphorylation and overall expression of NDRG1 in MDA-MB-231 breast cancer cells Breast cancer cells were treated for different times with MNK-I1 (5 μM). Levels of specific proteins in **(A)** MDA-MB-231 and **(B)** MDA-MB-453 cells were analysed by Western blot with indicated antibodies (the latter display two distinct bands for P-eIF4E). Ratio of p-NDRG1/NDRG1 was calculated using Image J. NDRG1 mRNA levels in **(C)** MDA-MB-231 and **(D)** MDA-MB-453 cells were analysed by RT-qPCR. **(E)** MDA-MB-231 cells were pretreated with MNK-I1 (5 μM) for 4 h and followed by 100 ng/ml actinomycin D for 16 h, where indicated. NDRG1 mRNA were analysed by qPCR. Data are shown as mean ± S.E.M. from three replicates. *P < 0.05; **P < 0.01; ***P < 0.001; ****P < 0.0001.

Treatment of MDA-MB-231 or -453 (‘triple-negative’ metastatic) or MCF7 (steroid receptor-positive) cells with the potent and specific MNK inhibitor MNK-I1 [9] decreased the phosphorylation of NDRG1 by 8 h. In MDA-MB-231 cells, phosphorylated NDRG1 levels recovered again by 24 or 48 h. MNK-I1 treatment also increased the levels of NDRG1 protein (Figure 1A). Taking this into account, it is clear that MNK-I1 still suppresses the phosphorylation of NDRG1 (normalised to total NDRG1) at later times, but less effectively than at 8 h (Figure 1A). The recovery in P-NDRG1 levels and increase in total NDRG1 upon extended treatment with MNK-I1 were also observed in MDA-MB-453 (Figure 1B) but not in MCF7 cells (Supplementary Figure 1A). Importantly, MNK-I1 had no effect on the levels of the SGK1 protein in either cell line, ruling out that its effect on P-NDRG1 are mediated through changes in SGK1 levels (Figure 1A,1B). MNK-I1 did cause a slight increase in total MNK1 protein levels in MDA-MB-453 cells (Figure 1B) but not in MDA-MB-231 cells (Figure 1A); the reason for this is unclear.

Interestingly, two bands are observed for P-eIF4E in lysates from MDA-MB-453 cells (Figure 1B); since both are eliminated by MNK-I1, they presumably correspond to different species of eIF4E. However, only one band is seen with the ‘total’ eIF4E antibody; this may reflect the existence of two isoforms of eIF4E in these cells. Since the phosphorylation site is at the extreme C-terminus [12] of eIF4E, the shorter of these species must presumably lack or possess a different N-terminus.

### MNKs regulate the expression of the NDRG1 mRNA

Treating MDA-MB-231 or -453 cells with MNK-I1 increased the levels of *NDRG1* mRNA (assessed by RT-qPCR; Figure 1C,1D), an effect which roughly paralleled the increase in NDRG1 protein (Figure 1A,1B). In contrast, treating MCF7 cells with MNK-I1 did not affect *NDRG1* mRNA levels (Supplementary Figure 1B).

MNK-I1's ability to increase *NDRG1* mRNA levels could be mediated through altered stability of this mRNA. To assess this, MDA-MB-231 cells were treated with MNK-I1 for 4 h and then, in some cases, with actinomycin D for a further 16 h to block mRNA transcription. As expected, actinomycin D blocked the marked increase in *NDRG1* mRNA levels caused by MNK-I1 (Figure 1E). In the presence of actinomycin D, *NDRG1* mRNA levels fell markedly, and similarly in the presence or absence of MNK-I1. These data show that the *NDRG1* mRNA is unstable and rules out that MNK-I1 increases *NDRG1* mRNA levels by stabilising it.

Thus, MNKs likely repress the expression of NDRG1 at the level of transcription. It is not clear why the effect differs between MDA-MB-231 and MDA-MB-453 cells; since p53 is linked to control of *NDRG1* expression [21, 22], it may reflect that fact that p53 protein levels are much higher in the former than in the latter (data not shown).

### The MNKs modulate the phosphorylation of NDRG1

**Figure 2:**
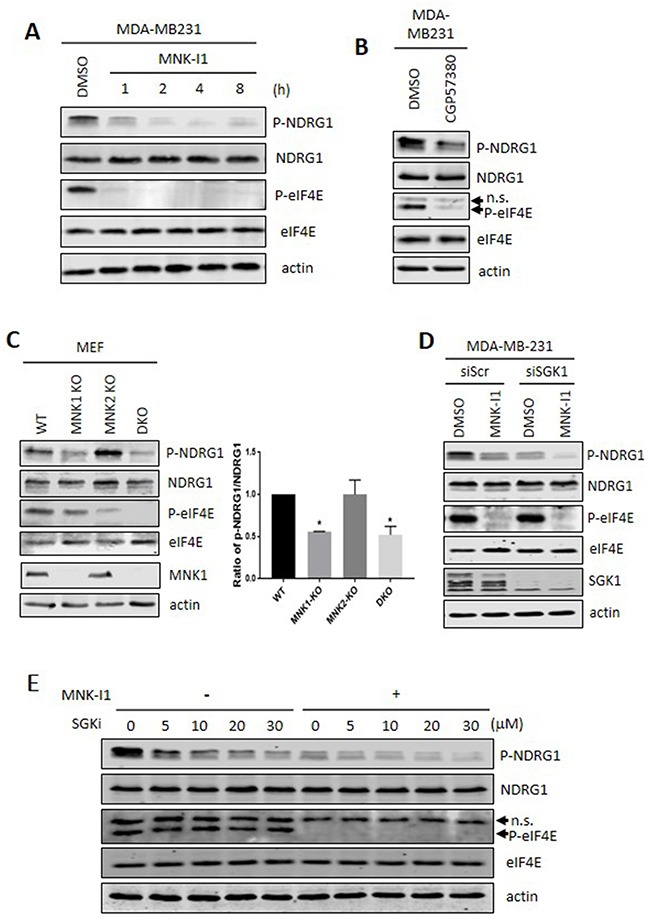
Pharmacological and genetic evidence that MNKs modulate the phosphorylation of NDRG1 **(A)** MDA-MB-231 cells were treated with 5 μM MNK-I1 for the indicated times and lysates were then analysed by Western blot. **(B)** MDA-MB-231 cells were treated with 25 μM CGP57380 for 2 h. Lysates were analysed by Western blot. **(C)** Lysates from wild type, MNK1-KO, MNK2-KO, and MNK1+MNK2 double knockout (DKO) MEFs were analysed by immunoblot with the indicated antibodies. Ratio of p-NDRG1/NDRG1 was calculated using Image J. Data are shown as mean ± S.E.M. from three replicates. *P < 0.05. **(D)** SGK1 was knocked down by an siRNA in MDA-MB-231 cells and, where shown, cells were then treated with 5 μM MNK-I1 for 4 h. Lysates were analysed by Western blot. **(E)** MDA-MB-231 cells were treated with increasing concentrations of SGK inhibitor (SGKi) together with 5 μM MNK-I1, where indicated, for 4h. Lysates were analysed by Western blot. In some cases, the antibody used for P-eIF4E recognises an additional band which appears to be a non-specific reaction and is not affected by the MNK inhibitors (indicated as ‘n.s.’ with an arrowhead in panels **B** and **E**).

Treatment of MDA-MB-231 cells with MNK-I1 for shorter times, up to 8h, (Figure 2A) caused a rapid decrease in the phosphorylation of NDRG1 (the effect already being maximal by 2 h). This is consistent with their effects being mediated through inhibition of the MNKs, rather than rapid down-regulation of another kinase acting on NDRG1. At times between 8 and 24 h, total and phosphorylated NDRG1 followed the expected trend based on the data for 8 and 24 h (not shown).

To study this further, we used another small molecule MNK inhibitor, CGP57380. This was the first MNK inhibitor to be reported [23] but is weaker than MNK-I1 [9]. Nonetheless, CGP57380 partially inhibited P-eIF4E and P-NDRG1 (Figure 2B).

To confirm the role of the MNKs in the phosphorylation of NDRG1, we employed cells in which one or both MNKs have been ‘knocked out’ [24]. NDRG1 phosphorylation was readily detected in mouse embryonic fibroblasts (MEFs) from wild-type mice but was much lower in cells from MNK1+MNK2 double-knockout (DKO) animals (Figure 2C). Knocking out MNK1 sharply decreased NDRG1 phosphorylation while, in contrast, MNK2 knock-out did not (although it did reduce P-eIF4E and did not cause an increase in MNK1 protein levels). perhaps because, unlike MNK1, it is basally active [10]. Taken together, these data, provide independent genetic evidence that MNKs modulate NDRG1 phosphorylation and show that it is primarily MNK1, which regulates NDRG1 phosphorylation. Lastly, the differing data for P-eIF4E and P-NDRG1 in single MNK-KO cells show that the effect of impairing MNK activity on NDRG1 phosphorylation is not a consequence of decreased phosphorylation of eIF4E.

To test the contribution of SGK1 to phosphorylation of NDRG1, we used siRNA to deplete SGK1 (Figure 2D). This markedly decreased P-NDRG1, but a residual level did remain. MNK-I1 treatment almost completely eliminated the remaining P-NDRG1 (Figure 2D), indicating that MNKs and SGK1 make independent inputs to phosphorylation of the same subset of sites in NDRG1.

To further assess the contributions of SGK1 and MNKs to controlling NDRG1 phosphorylation, we tested the effect of the SGK inhibitor SGKi (GSK650394; [25]) at a range of concentrations, with or without MNK-I1, (Figure 2E). At all SGKi concentrations, MNK-I1 further inhibited P-NDRG1. This is not because SGKi inhibits MNK function as it did not inhibit P-eIF4E showing that SGKi and MNKs make separate inputs to phosphorylation of NDRG1. It is also not because SGK2 provides the residual phosphorylation of NDRG1 as this compound inhibits both SGK1 and SGK2 [25].

### Roles of SGK1 and MNKs in modulating the phosphorylation of NDRG1

**Figure 3:**
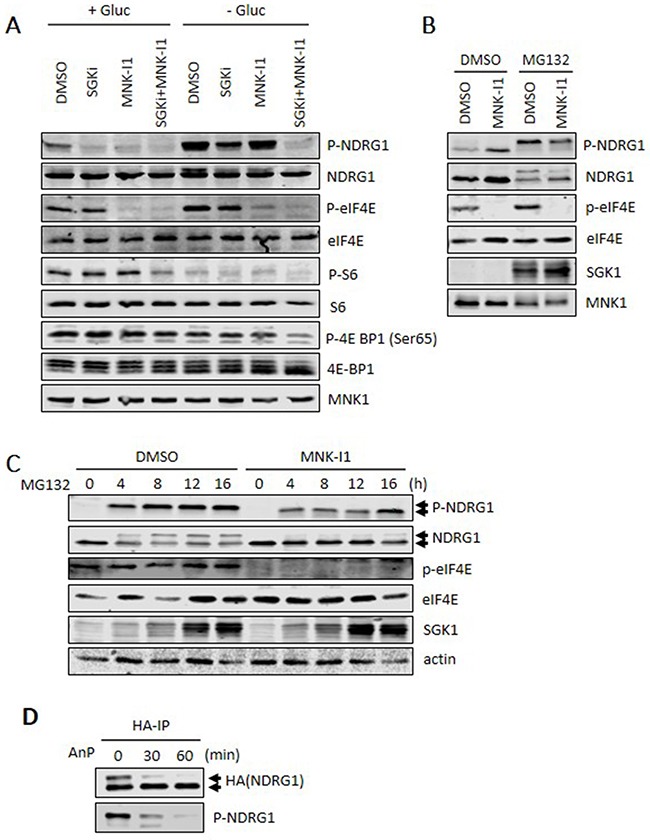
Roles of SGK1 and MNKs in modulating the phosphorylation of NDRG1 **(A)** Where indicated, MDA-MB-231 cells were starved of glucose for 4 h in the presence, where shown, of 5 μM MNK-I1 and/or 10 μM SGKi. Lysates were analysed by Western blot. **(B)** MDA-MB-231 cells were treated with 5 μM MG132 and 5 μM MNK-I1 for 16 h and lysates were analysed by Western blot. **(C)** MDA-MB-231 cells were pretreated with 5 μM MNK-I1 for 1 h and then with 5 μM MG132 for the indicated times. Cell lysates were analysed by Western blot. **(D)** MDA-MB-231 cells were transfected with a vector for HA-tagged NDRG1 and then treated with 5 μM MG132 and 5 μM MNK-I1 for 12 h. HA-IP was performed to isolate NDRG1. Beads were then incubated with Antarctic phosphatase (AnP) at 37°C for 30 or 60 min. Reaction products were analysed by Western blot.

In the absence of glucose, NDRG1 phosphorylation increases (Figure 3A). When used alone, neither an SGK inhibitor (SGKi) nor MNK-I1 could block phosphorylation of NDRG1, although, when used together, they almost completely eliminated it (Figure 3A), again showing that SGK1 and MNK1 work in parallel to promote NDRG1 phosphorylation. Interestingly, in glucose starved cells, but not controls, the combination of SGKi and MNK-I1 caused a shift in the migration of 4E-BP1 to faster moving, less phosphorylated forms, and a decrease in the phosphorylation of ribosomal protein S6. Both are regulated by mTORC1 signalling. This suggests that SGKi and MNK-I1 together, but not individually, decrease in mTORC1 signalling (Figure 3A). This may reflect, at least in part, the recently reported input from MNKs to mTORC1 signalling [26]. The levels of MNK1 protein were not affected by glucose starvation, of by SGKi and/or MNK-I1.

It should be noted that, since SGKi also inhibits SGK2 [25], and as SGK1 primes NDRG1 for phosphorylation by glycogen synthase kinase-3 (GSK3; [2]), effects of this compound on the behaviour of NDRG1 may reflect interference with one or both of these inputs.

Treating cells with the proteasome inhibitor MG132 greatly increased SGK1 levels (see also Figure 3B,3C) and, presumably as a consequence, P-NDRG1. This reflects the fact that SGK1 is degraded via a proteasome-dependent pathway, which is blocked by this compound [27, 28]). MG132 also caused the appearance of a slower-migrating form of NDRG1 but did not affect MNK1 levels (Figure 3B). The slower-migrating band was heavily phosphorylated (Figure 3B,3C). MNK-I1 decreased the proportion of the upper band (Figure 3C) showing its appearance depends on MNK1 activity, presumably reflecting one or more MNK-dependent phosphorylation event(s). To test this, HA-NDRG1 was immunoprecipitated from MG132-treated cells, and then incubated with Antarctic phosphatase (AnP). This completely eliminated the upper band (and the signal for P-NDRG1; lower part of Figure 3D) indicating the mobility shift is indeed due to phosphorylation and providing further evidence that MNKs promote NDRG1 phosphorylation.

### MNK1 does not directly phosphorylate NDRG1 or SGK1

**Figure 4:**
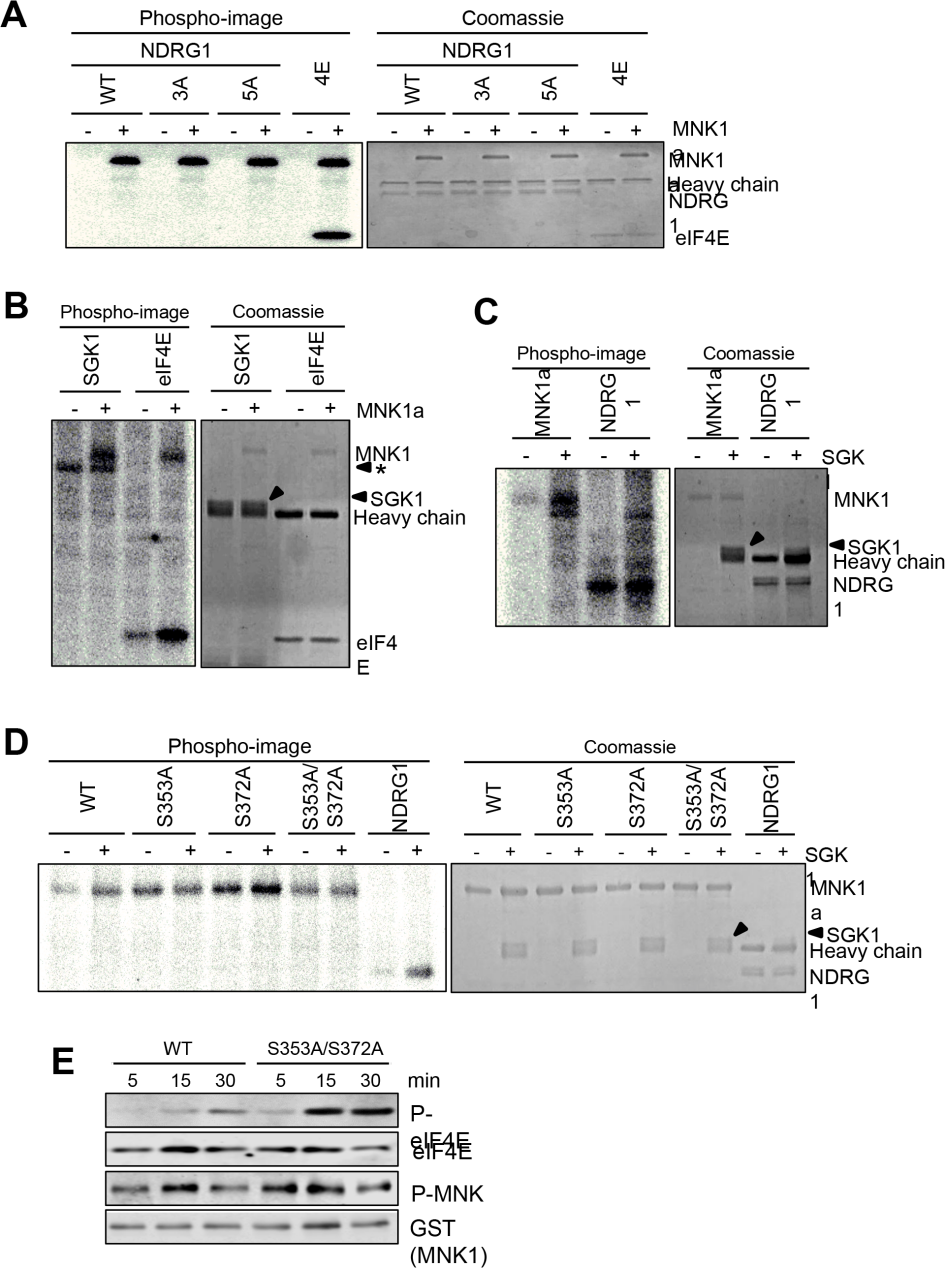
SGK1 phosphorylates MNK1 *in vitro* Kinase assay using [γ-^32^P]ATP. **(A)** Immunoprecipitates of wild-type NDRG1 or the indicated mutants (see Methods for details) or **(B)** immunoprecipitated SGK1 were incubated with or without purified MNK1a at 30°C for 30 min. Incorporated radiolabel was assessed by phosphorimager (left). Protein levels were assessed by Coomassie blue staining (right). eIF4E was used as a positive control for MNK1a activity. * denotes a non-specific band in the SGK1 immunoprecipitate. **(C)** Kinase assays using [γ-^32^P]ATP were performed with human MNK1a with or without SGK1′. and beads (with/without SGK1) on the left (phosphorimage), and protein levels were assessed by Coomassie blue staining (on the right). NDRG1 was used as a positive control for SGK1 activity. **(D)** Kinase assay using [γ-^32^P]ATP was used to test the ability of SGK1 to phosphorylate purified MNK1a protein and the indicated mutants. NDRG1 was used as a positive control. In **(C)** and **(D)**, +SGK1 indicates that SGK1was immunoprecipitated from cells transfected with a vector encoding SGK1. –SGK1 indicates mockimmunoprecipitaion from non-transfected cells.’ **(E)** The activity of activated MNK1a protein was assessed using non-radioactive ATP with eIF4E as substrate and samples were withdrawn at the indicated times. Samples were analysed by SDS-PAGE/immunoblot, using the indicated antibodies.

Taken together, the above data indicate that MNK1 positively regulates NDRG1 phosphorylation. To test whether MNK1 directly phosphorylates NDRG1, we incubated active recombinant MNK1, expressed in HEK293 cells and purified on GSH beads, with HA-tagged wild-type or mutant NDRG1 and radioactive [g-^32^P]ATP. MNK1 clearly phosphorylated purified recombinant eIF4E, a positive control. However, MNK1 did not phosphorylate HA-NDRG1 or (as potential controls) mutants of that protein where certain known phosphorylation sites had been altered to non-phosphorylatable alanine residues (Figure 4A), although Coomassie staining confirmed that similar amounts of HA-tagged NDRG1 (wild-type and mutant) and HA-tagged eIF4E were used (Figure 4A).

MNK1 could not phosphorylate SGK1, even in this sensitive type of radioactive assay (Figure 4B). It is formally possible that the effects of MNK1 on P-NDRG1 are mediated through the related protein SGK2.

### MNK1 is a substrate for SGK1 *in vitro*

Given the above connections between SGK1 signalling and MNK1, we tested whether SGK1 could phosphorylate MNK1. In at least four independent experiments, SGK1 reproducibly catalysed the incorporation of radiolabel into GST-MNK1a purified from HEK293 cells (Figure 4C), above the background phosphorylation. Inspection of the sequences of MNK1a revealed two potential phosphorylation sites for SGK1 (Ser353 and Ser372), in a region that is absent from MNK1b. Its sequence is QV***L***Q***R***NS**S**TMDLTLFAAEAIA***L***N***R***QL**S**QHEENELA, where potential SGK1 target sites are shown bold/underlined and residues corresponding to the SGK1 recognition motif (LxRxxS/T [29]) are bold/italicised. Both sites are conserved, along with the main features of the adjacent sequence, in mouse MNK1, but not in human MNK2a or mouse MNK2.

We created mutants in which Ser353 or Ser372, or both, were converted to non-phosphorylatable alanine residues. Whereas wild-type MNK1 and MNK1 [S372A] each underwent greater radiolabelling when incubated with SGK1, this was not the case for the S353A or S353A/S372A mutants (Figure 4D). Thus, SGK1 phosphorylates MNK1 in a conserved C-terminal site, S353. This site has been reported to be phosphorylated *in vivo* (www.phosphosite.org).

Mutating the SGK1 sites increased MNK1a activity (Figure 4E), as shown by increased p-eIF4E but did not affect the level of phosphorylation of MNK1 in its activation loop. Similar data were reproducibly obtained for both the S353A/S372A double (Figure 4E) and the S353A mutants (not shown). This indicates that SGK1 provides an inhibitory input to MNK1 through phosphorylation in its C-terminus, and thus that SGK1 may negatively regulate eIF4E phosphorylation in cells where MNK1a is the major MNK isoform.

### Roles of MNKs and SGK1in regulating cell migration/invasion do not require NDRG1

NDRG1 is generally reported to repress migration or metastasis [30, 31, 32, 33], while MNKs promote migration of cancer cells such as MDA-MB-231 [9]. The finding that MNK inhibition increases NDRG1 protein levels raised the possibility that inhibiting MNK1 impairs cell migration through this effect. We therefore examined the effect of NDRG1 knock-down on the ability of MNK-I1 to inhibit cell migration.

**Figure 5:**
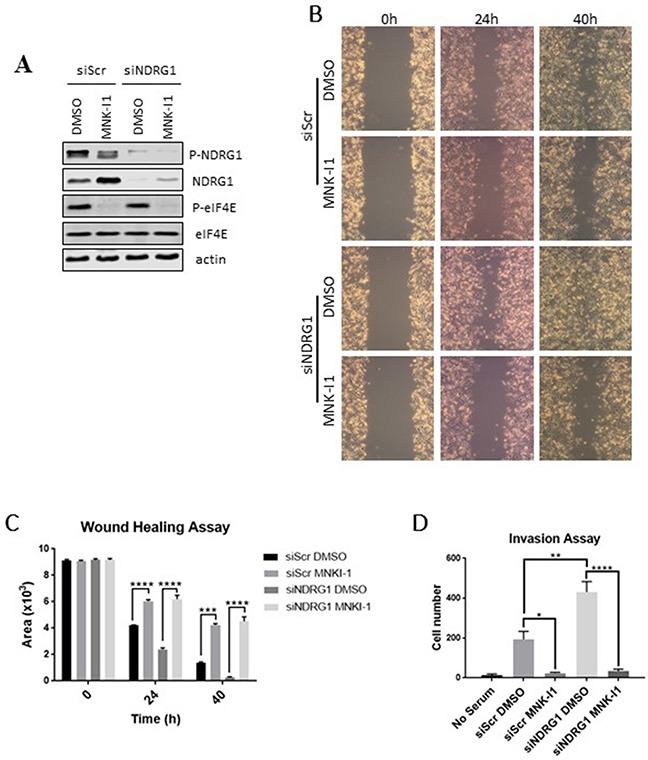
The regulation of cell migration by the MNKs does not require NDRG1 NDRG1 was knocked down by an siRNA in MDA-MB-231 cells and cells were treated with 5 μM MNK-I1. A scrambled siRNA (siScr) was used as a negative control. **(A)** Cell lysates were analysed by Western blot. Wound healing was tested in MDA-MB-231 cells in the presence of MNK-I1 (5 μM) where indicated. **(B)** Photographs were taken under a light microscope and **(C)** quantified using ImageJ. **(D)** A cell invasion assay was performed to measure the invasive ability of these cells. Data are shown as mean ± S.E.M. from three replicates. *P < 0.05; **P < 0.01; ***P < 0.001; ****P < 0.0001.

Knockdown of NDRG1 was efficient (Figure 5A) and, as expected from the data in Figure 1A,1B, the MNK-I1 increases NDRG1 levels. Knockdown of NDRG1 enhanced cell migration, confirming that NDRG1 normally impairs this process (Figure 5B; quantified in Figure 5C). MNK-I1 inhibited migration of MDA-MB-231 cells to almost identical extents whether or not NDRG1 had been knocked down (Figure 5B,5C), indicating that MNK inhibition impairs migration independently of NDRG1. Thus, while regulation of NDRG1 protein levels by MNKs probably contributes to the MNKs’ effects on cell migration, MNK-I1 also affects migration through additional mechanisms. This is consistent with the observation that, in MEFs, knocking out either MNK1 or MNK2 partially impairs cell migration [9], while only MNK1 plays a role in modulating NDRG1 phosphorylation (Figure 2C).

Knocking down NDRG1 also promoted invasion of MDA-MB-231 cells (Figure 5D). MNK-I1 almost completely blocked invasion in control and NDRG1-knockdown cells, similar to the situation for the effects of these manipulations on cell migration.

### SGK1 affects the increased breast cancer cell migration induced by NDRG1 deficiency

**Figure 6:**
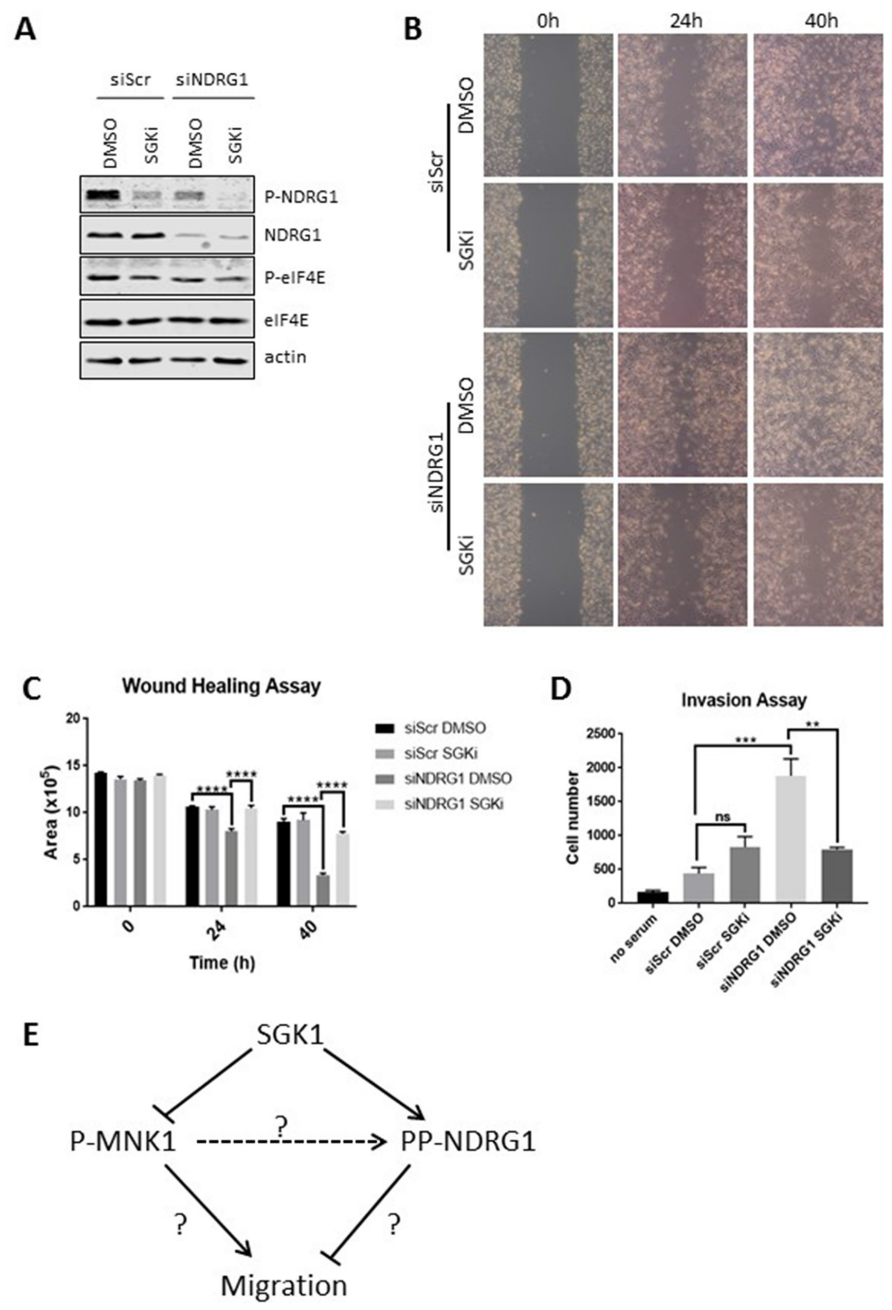
SGK1 inhibition impairs cell migration induced by NDRG1 knock-down NDRG1 was knocked down by an siRNA in MDA-MB-231 cells and cells were treated with 10 μM SGKi. A scrambled siRNA (siScr) was used as a negative control. **(A)** Cell lysates were analysed by Western blot. Wound healing was tested in MDA-MB-231 cells with or without the SGK inhibitor. **(B)** Photos were taken under a light microscope and **(C)** quantified with ImageJ. **(D)** Invasion assay was performed on MDA-MB-231 cells with/without SGK inhibitor. Data are shown as mean ± S.E.M. from three replicates. **P < 0.01; ***P < 0.001; ****P < 0.0001. **(E)** Novel links between MNK1, SGK1 and NDRG1 uncovered by this study. ‘?’ indicates links which are indirect or remain unclear. The dashed line indicates that MNK1 does not directly phosphorylate NDRG1. Please see the text for further details.

Given the potential link between SGK1, NDRG1 phosphorylation and cancer cell migration we used siRNA to deplete NDRG1 in MDA-MB-231 cells and then treated them with SGKi (Figure 6A). As already noted (Figure 5B,5C), knock-down of NDRG1 enhanced cell migration. Interestingly, whereas SGKi did not affect the wound-healing ability of MDA-MB-231 cells under control conditions, it did block the increased migration that is induced by NDRG1 knock-down (Figure 6B,6C).

NDRG1 knock-down also enhanced cell invasion and, similar to the situation for cell migration, SGKi inhibited invasion by NDRG1-depleted but not control cells (Figure 6D). These findings are consistent with data showing that overexpressing or knocking out SGK1 can, respectively, accelerate or impede cell migration [34, 35], but shows that the ability of SGK inhibition to control cell migration depends critically on the levels of NDRG1. Thus, it is important to know the prevailing levels of NDRG1 protein, since where they are high, SGK inhibition will likely be ineffective in impairing migration or invasion. In contrast, the ability of MNK inhibition to affect migration or invasion is independent of the levels of NDRG1. Our data thus indicate that SGK1 and the MNKs affect cell migration in distinct ways.

## DISCUSSION

Our data reveal that MNKs regulate both the cellular levels and the phosphorylation of the metastasis suppressor NDRG1. MNK inhibition impairs cell migration and invasion, while deleting NDRG1 increases these processes. Thus, it is likely that the control of NDRG1 protein levels by the MNKs contributes to their role in regulating cell migration/invasion. However, since MNK inhibition still impairs migration of cells depleted for NDRG1, the MNKs must also control migration via additional mechanisms. Figure 6E summarises the links between MNKs, SGK1 and NDRG1 revealed by our data.

MNK inhibition increases *NDRG1* mRNA levels, especially in MDA-MB-231 cells. Our data indicate that this reflects increased transcription of the *NDRG1* gene, adding to the emerging but still limited information indicating that MNKs regulate gene transcription. For example, eIF4E phosphorylation controls the transcriptional activity of NF-kB [36], indirectly via translational control of expression of IkBa. The *NDRG1* gene is not a target for NF-kB. Instead, it is controlled by other factors including p53, hypoxia-inducible factor 1a, AP1 and Egr-1 (reviewed [37]). Since none of them is known to be controlled by the MNKs, MNKs presumably control *NDRG1* expression by a novel mechanism; further studies are needed to understand this.

SGK1 does not affect cell migration under normal conditions although inhibition of SGK1 does block the faster migration of MDA-MB-231 cells that is induced by knocking down NDRG1. This suggests that SGK1 regulates other proteins which influence cell migration. It will be important to identify the substrates for SGK1 and the MNKs which mediate their effects on cell migration.

Our data also show that SGK1 can phosphorylate MNK1 at Ser353 *in vitro* and suggest that this phosphorylation event inhibits MNK1. The interplay between NDRG1, SGK1 and MNK1 is therefore quite complex (Figure 6E). One implication of the data is that SGK1 does not promote NDRG1 phosphorylation by activating MNK1, underscoring the conclusion that SGK1 and MNK1 make independent inputs to P-NDRG1.

Lastly, the use of NDRG1 phosphorylation as an indicator of the activity status of Akt/PKB and SGK1 [38], or their common upstream regulators, mTORC2 and phosphoinositide signalling [39], must take into account this additional input from MNKs (mainly MNK1). MNK1 does not phosphorylate NDRG1 or SGK1 directly and the mechanism by which it affects NDRG1 phosphorylation remains to be clarified.

Here we also observed that glucose starvation led to increased phosphorylation of NDRG1 which is reduced by SGKi, implying it is mediated by SGK1 and/or 2. The reason for this effect of glucose starvation is unclear; glucose starvation can elicit oxidative stress [40], which in turn inactivates the lipid phosphatase PTEN [41]. As PTEN opposes the signalling pathway that lies upstream of SGK1 [42], this may provide a mechanism to explain the increased phosphorylation of NDRG1 seen in glucose-starved cells.

In summary, we describe a novel target for regulation by MNK1, i.e., NDRG1, which also provides the first example of a protein which is regulated by one MNK but by not the other. We have therefore identified novel links between the oncogenic MAP kinase and PI 3-kinase/mTORC2 signalling pathways, which control MNK1 and SGK1, respectively.

The very recent development [43] of an MNK inhibitor which exerts anti-tumour effects *in vivo* points to the importance of understanding more fully the roles of MNKs in solid tumour biology. This study makes a substantial contribution to achieving that aim. Our data show that the ability of SGK inhibition to impair cell migration depends on the NDRG1 expression levels in the cell, whereas inhibiting the MNKs blocks cell migration and invasion irrespective of the NDRG1 levels. Thus, an important conclusion from our studies is that MNK inhibitors may be more widely applicable for tackling tumour metastasis than inhibitors of SGK1.

## MATERIALS AND METHODS

### Cell culture and treatment

Primary mouse embryonic fibroblasts (MEFs) were prepared from 13.5 day-old embryos and grown in DMEM plus 10% (v/v) fetal bovine serum (FBS); all experiments using them employed cells passaged *<five* times. Cells were grown in Dulbecco's modified Eagle's medium (DMEM, Invitrogen), containing 10% (v/v) FBS and maintained at 37°C in humidified air with 5% CO_2_.

Cells were lysed inlysis buffer (25 mM Tris-HCl [pH 8.0], 50 mM KCl, 50 mM β-glycerophosphate, 0.2 mM EDTA, 1% Triton X-100), containing 0.5 mM NaVO_3_, 0.1% 2-mercaptoethanol and protease inhibitors (Roche).

The MNK inhibitor, MNK-I1, was described earlier [44]. GSK650394 (SGKi, Sigma; [25]) was used to inhibit SGK1. Drugs were dissolved in DMSO and control cells received this vehicle.

### Gene silencing by small interfering RNA

Expression of *NDRG1* and *SGK1* was transiently knocked down with small-interfering RNAs (siRNA) in MDA-MB-231 cells. siRNAs were transfected into cells using Lipofectamine™ 3000 Transfection Reagent (Life Technologies). A scrambled siRNA was used as negative control. siRNAs for gene silencing: NDRG1: 5′-CAUCGAGACUUUACAUGGCUCUGUU-3′; SGK1: 5′-GGAGCUGUCUUGUAUGAGA-3′.

### Gel electrophoresis and immunoblotting

Cell lysate proteins were separated by 12.5% SDS-PAGE gel and transferred to nitrocellulose membranes (Bio-Rad). Membranes were blocked in 5% (w/v) fat-free milk powder at room temperature for 1 h and incubated with indicated primary antibodies (at 1:1000 dilution). Primary antibodies for p-NDRG1 (Thr346/356/366) (#5482), p-S6 (Ser240/244) (#2215), p-4E-BP1 (Ser65) (#9451), SGK1 (#12103) and MNK1 (#2195) eIF4E (#9742) were from Cell Signaling Technology. Antibodies for NDRG1 were from the Division of Signal Transduction Therapy, University of Dundee, for p-eIF4E Ser 209 from Millipore (07-823), the antibody for 4E-BP1 was reported in [11]) and antibodies for actin and HA were purchased from Sigma-Aldrich.

Immunoreactive bands were detected by fluorophore-conjugated secondary antibodies (ThermoFisher Scientific) and scanned by Li-Cor Odyssey^®^ imager.

### Cell transfection, mutagenesis and vectors

HEK293 cells were transfected using the calcium phosphate method. MDA-MB-231 cells were transfected using Lipofectamine 3000.

Vectors for GST-MNK1a [45], HA-tagged wild-type NDRG1 or the HA-NDRG1-Thr346/356/366A (3A) or HA-NDRG1-Thr328A/Ser330A/Thr346/356/366A (5A) [46] have been described previously. GST-MNK1a-Ser353A and Ser372A were made from GST-MNK1a WT, while HA-NDRG1-Thr 346/356/366A (3A) were made from HA-NDRG1-5A by mutagenesis, using the QuikChange procedure. Primers for QuikChange-mutagenesis: MNK1a-S353A forward: 5′-CTCCAGAGGAACAGCGCCACAATGGACCTGACG-3′, reverse: 5′-CGTCAGGTCCATTGTGGCGCTGTTCCTCTGGAG-3′; MNK1a-S372A forward: 5′-CCTTAACCGCCAGCTAGCTCAGCACGAAGAGAAC-3′, reverse: 5′-GTTCTCTTCGTGCTGAGCTAGCTGGCGGTTAAGG-3′; NDRG1-3A forward: 5′-ATGCGGTCCCGCACAGCCTCTGGCTCCAGTGTC-3′, reverse: 5′-GACACTGGAGCCAGAGGCTGTGCGGGACCGCAT-3′.

### Immunoprecipitations (IPs) for HA or SGK1

Six μg of vector was transfected into 10cm plate of HEK 293 cells. Cell lysate (500 μg) was incubated with 2 μl HA or SGK1 antibody in lysis buffer containing 0.5 mM NaVO_3_, 0.1% 2-mercaptoethanol and protease inhibitors (Roche) at 4°C for 2 h. 20 μl pre-washed protein G beads (GE Healthcare) were added into the mixture and incubated at 4°C for another 2 h with gentle agitation. The beads were washed twice with high-salt buffer (50 mM Tris-HCl [pH7.5], 50 mM β-glycerophosphate, 350 mM NaCl, 1 mM MgCl_2_, 0.5 mM EDTA, 0.1 mM EGTA, 1% Triton X-100). All immunoprecipitates were washed again with kinase assay buffer (lacking radioactive ATP) twice more prior to assay. For SGK1 IP, cells were pretreated with 10 μM MG132 to stabilize SGK1 for 16 h before harvesting.

### GST pull-down and MNK1 purification

Six μg of GST-tagged-MNK1 vector was transfected into HEK293 cells in 10 cm plates. Whole cell lysate was harvested from cells using lysis buffer containing 0.1% mercaptoethanol, 0.5 mM NaVO_3_ and protease inhibitors (Roche). Whole cell lysate was incubated with 300 μl of GST beads at 4°C for 2 h. The beads were then centrifuged at 6,000 rpm for 30 s and washed twice in high-salt buffer and a further two times in GST-purification washing buffer (25 mM Tris-HCl, pH 7.5; 50 mM KCl; 5 mM 2-mercaptoethanol). Protein was then eluted from GST-beads by incubating with 300 μl elution buffer (washing buffer containing 40 mM reduced glutathione) on ice for 15 min. Elution was repeated twice more. The samples from each step were kept and analysed by SDS-PAGE.

The protein eluate was then dialysed using Slide-A-Lyzer dialysis cassettes (Thermo Scientific) in dialysis buffer at 4°C overnight. Amounts of protein were estimated by running them and known amounts of bovine serum albumin on SDS-PAGE followed by staining with Coomassie brilliant blue. The purified proteins were divided into aliquots and immediately stored at -80°C.

Activated MNK-1 was purified from cells which were placed in serum-free medium overnight, and treated with 1 μM phorbol myristate acetate for 30 min, to activate MNK1.

### RT-qPCR analysis

Total RNA was extracted from cells using Trizol (Sigma). Total RNA (1 μg) from each sample was used for cDNA synthesis using QuantiNova Reverse Transcription Kit (QIAGEN). T-PCR was performed using cDNA as template and Fast SYBR Green master mix (Applied Biosystems) on an Applied Biosystems detection system. Primers for qRT-PCR: *NDRG1* forward: 5′-GGCAACCTGCACCTGTTCATCAAT-3′, reverse: 5′-TGAGGAGAGTGGTCTTTGTTGGGT-3′; *SGK1* forward: 5′-CTCAGTCTCTTTTGGGCTCTTT-3′, reverse: 5′-TTTCTTCTTCAGGATGGCTTTC-3′. The relative amount of mRNA was normalized to *GAPDH*.

### Wound healing, migration and invasion assays

These assays were performed as described previously [9].

Transwell migration/invasion assays were performed using 24-well 6.5 mm diameter inserts with an 8.0 μm pore size (Costar). Cells were serum-starved overnight and pretreated with 5 μM MNK-I1 or 10 μM SGKi for 1 h before the assay.

The top of the Transwell insert was coated with 0.2 mg/ml gelatin (Sigma) at 37°C for 2 h. 1.5×10^4^ MDA-MB-231 cells (resuspended in 200 μL serum-free DMEM) were added to the top of the insert. Cells were incubated at 37°C and migration was assessed after 24 h. Medium was removed from the top and bottom of the wells; cells on the insert were fixed with 3.7% formaldehyde for 2 min followed by 20 min with 100% methanol. Cells were stained with 4′,6-diamidino-2-phenylindole (DAPI; 5 μg/μl) for 15 min, protected from light, washed twice in PBS and cells on the top were removed with a cotton stick. At least five photographs were acquired for each sample using a fluorescent microscope (Nikon) and the number of cells was analysed using Image J software.

### *In vitro* protein kinase assays

Recombinant glutathione *S*-transferase (GST)-MNK fusion proteins were purified by glutathione pull-down from lysates of appropriately-transfected HEK293 cells.

SGK1 protein was prepared by immunoprecipitation using anti-SGK1 antibodies. For kinase assay with [γ-^32^P]ATP, beads with SGK1 protein and 300 ng non-activated MNK were incubated with 0.1 μl [γ-^32^P]ATP (where used), 3000 Ci/mmol (PerkinElmer) and 0.2 μl non-radioactive ATP (10 μM) (Sigma) in kinase assay buffer (25 mM Tris-HCl pH7.5, 50 mM KCl and 2 mM MgCl_2_) at 30°C for 30 min. Where indicated, HA-NDRG1 (immunoprecipitated from HEK293 cell lysates) or 250 ng eIF4E (expressed in *E. coli*) were used as test substrates. NDRG1 immunoprecipitates were washed twice with high salt buffer to remove contaminating kinase activity. Products were analysed by SDS-PAGE. Coomassie blue staining was used to assess protein levels and a phosphorimager scanner (PerkinElmer) to visualise the radioactive signals.

Non-radioactive assays were performed as described above, using the indicated substrates, but using only unlabelled ATP.

## SUPPLEMENTARY FIGURE



## Abbreviations

AnP: Antarctic phosphatase
DMEM: Dulbecco's modified Eagle's medium
eIF: eukaryotic initiation factor
ERK: Extracellular-signal Regulated Kinase
GST: Glutathione S-transferase
HA: hemagglutinin
HEPES: 4-(2-hydroxyethyl)-1-piperazineethanesulfonic acid
MNK: MAP kinase-interacting kinase (MNK)
MNK-I1: MNK inhibitor
mTORC2: mammalian Target of Rapamycin complex 2
NDRG1: N-myc downregulated gene 1
P-eIF4E: phosphorylated eIF4E
P-NDRG1: phosphorylated NDRG1
SGK1: serum and glucocorticoid-regulated kinase 1
SGKi: SGK1 inhibitor
siRNA: small interfering RNA

## References

[1] Fang BA, Kovacevic Z, Park KC, Kalinowski DS, Jansson PJ, Lane DJ, Sahni S, Richardson DR (2014). Molecular functions of the iron-regulated metastasis suppressor, NDRG1, and its potential as a molecular target for cancer therapy. Biochim Biophys Acta.

[2] Murray JT, Campbell DG, Morrice N, Auld GC, Shpiro N, Marquez R, Peggie M, Bain J, Bloomberg GB, Grahammer F, Lang F, Wulff P, Kuhl D (2004). Exploitation of KESTREL to identify NDRG family members as physiological substrates for SGK1 and GSK3. Biochem J.

[3] Liu W, Kovacevic Z, Peng Z, Jin R, Wang P, Yue F, Zheng M, Huang ML, Jansson PJ, Richardson V, Kalinowski DS, Lane DJ, Merlot AM (2015). The molecular effect of metastasis suppressors on Src signaling and tumorigenesis: new therapeutic targets. Oncotarget.

[4] Fotovati A, Abu-Ali S, Kage M, Shirouzu K, Yamana H, Kuwano M (2011). N-myc downstream-regulated gene 1 (NDRG1) a differentiation marker of human breast cancer. Pathol Oncol Res.

[5] Sun J, Zhang D, Bae DH, Sahni S, Jansson P, Zheng Y, Zhao Q, Yue F, Zheng M, Kovacevic Z, Richardson DR (2013). Metastasis suppressor, NDRG1, mediates its activity through signaling pathways and molecular motors. Carcinogenesis.

[6] Fukunaga R, Hunter T (1997). Mnk1, a new MAP kinase-activated protein kinase, isolated by a novel expression screening method for identifying protein kinase substrates. EMBO J.

[7] Waskiewicz AJ, Flynn A, Proud CG, Cooper JA (1997). Mitogen-activated kinases activate the serine/threonine kinases Mnk1 and Mnk2. EMBO J.

[8] Buxade M, Parra-Palau JL, Proud CG (2008). The Mnks: MAP kinase-interacting kinases (MAP kinase signal-integrating kinases). Front Biosci.

[9] Beggs JE, Tian S, Jones GG, Xie J, Iadevaia V, Jenei V, Thomas GJ, Proud CG (2015). The MAP kinase-interacting kinases regulate cell migration, vimentin expression and eIF4E/CYFIP1 binding. Biochem J.

[10] Scheper GC, Morrice NA, Kleijn M, Proud CG (2001). The MAP kinase signal-integrating kinase Mnk2 is an eIF4E kinase with high basal activity in mammalian cells. Mol Cell Biol.

[11] Wang X, Flynn A, Waskiewicz AJ, Webb BL, Vries RG, Baines IA, Cooper J, Proud CG (1998). The phosphorylation of eukaryotic initiation factor eIF4E in response to phorbol esters, cell stresses and cytokines is mediated by distinct MAP kinase pathways. J Biol Chem.

[12] Flynn A, Proud CG (1995). Serine 209, not serine 53, is the major site of phosphorylation in initiation factor eIF-4E in serum-treated Chinese hamster ovary cells. J Biol Chem.

[13] Pelletier J, Graff J, Ruggero D, Sonenberg N (2015). Targeting the eIF4F translation initiation complex: a critical nexus for cancer development. Cancer Res.

[14] Scheper GC, Morrice NA, Kleijn M, Proud CG (2001). The mitogen-activated protein kinase signal-integrating kinase Mnk2 is a eukaryotic initiation factor 4E kinase with high levels of basal activity in mammalian cells. Mol Cell Biol.

[15] Buxade M, Morrice N, Krebs DL, Proud CG (2008). The PSF.p54nrb complex is a novel Mnk substrate that binds the mRNA for tumor necrosis factor alpha. J Biol Chem.

[16] Buxade M, Parra JL, Rousseau S, Shpiro N, Marquez R, Morrice N, Bain J, Espel E, Proud CG (2005). The Mnks are novel components in the control of TNFalpha biosynthesis and phosphorylate and regulate hnRNP A1. Immunity.

[17] Proud CG (2015). Mnks, eIF4E phosphorylation and cancer. Biochim Biophys Acta.

[18] Furic L, Rong L, Larsson O, Koumakpayi IH, Yoshida K, Brueschke A, Petroulakis E, Robichaud N, Pollak M, Gaboury LA, Pandolfi PP, Saad F, Sonenberg N (2010). eIF4E phosphorylation promotes tumorigenesis and is associated with prostate cancer progression. Proc Natl Acad Sci U S A.

[19] Ueda T, Sasaki M, Elia AJ, Chio II, Hamada K, Fukunaga R, Mak TW (2010). Combined deficiency for MAP kinase-interacting kinase 1 and 2 (Mnk1 and Mnk2) delays tumor development. Proc Natl Acad Sci U S A.

[20] Diab S, Kumarasiri M, Yu M, Teo T, Proud C, Milne R, Wang S (2014). MAP kinase-interacting kinases-emerging targets against cancer. Chem Biol.

[21] Stein S, Thomas EK, Herzog B, Westfall MD, Rocheleau JV, Jackson RS, Wang M, Liang P (2004). NDRG1 is necessary for p53-dependent apoptosis. J Biol Chem.

[22] Croessmann S, Wong HY, Zabransky DJ, Chu D, Mendonca J, Sharma A, Mohseni M, Rosen DM, Scharpf RB, Cidado J, Cochran RL, Parsons HA, Dalton WB (2015). NDRG1 links p53 with proliferation-mediated centrosome homeostasis and genome stability. Proc Natl Acad Sci U S A.

[23] Tschopp C, Knauf U, Brauchle M, Zurini M, Ramage P, Glueck D, New L, Han J, Gram H (2000). Phosphorylation of eIF-4E on Ser 209 in response to mitogenic and inflammatory stimuli is faithfully detected by specific antibodies. Mol Cell Biol Res Commun.

[24] Ueda T, Watanabe-Fukunaga R, Fukuyama H, Nagata S, Fukunaga R (2004). Mnk2 and Mnk1 are essential for constitutive and inducible phosphorylation of eukaryotic initiation factor 4E but not for cell growth or development. Mol Cell Biol.

[25] Sherk AB, Frigo DE, Schnackenberg CG, Bray JD, Laping NJ, Trizna W, Hammond M, Patterson JR, Thompson SK, Kazmin D, Norris JD, McDonnell DP (2008). Development of a small-molecule serum- and glucocorticoid-regulated kinase-1 antagonist and its evaluation as a prostate cancer therapeutic. Cancer Res.

[26] Brown MC, Gromeier M (2017). MNK controls mTORC1: substrate association through regulation of TELO2 binding with mTORC1. Cell Rep.

[27] Brickley DR, Mikosz CA, Hagan CR, Conzen SD (2002). Ubiquitin modification of serum and glucocorticoid-induced protein kinase-1 (SGK-1). J Biol Chem.

[28] Heise CJ, Xu BE, Deaton SL, Cha SK, Cheng CJ, Earnest S, Sengupta S, Juang YC, Stippec S, Xu Y, Zhao Y, Huang CL, Cobb MH (2010). Serum and glucocorticoid-induced kinase (SGK) 1 and the epithelial sodium channel are regulated by multiple with no lysine (WNK) family members. J Biol Chem.

[29] Murray JT, Cummings LA, Bloomberg GB, Cohen P (2005). Identification of different specificity requirements between SGK1 and PKBalpha. FEBS Lett.

[30] Bandyopadhyay S, Wang Y, Zhan R, Pai SK, Watabe M, Iiizumi M, Furuta E, Mohinta S, Liu W, Hirota S, Hosobe S, Tsukada T, Miura K (2006). The tumor metastasis suppressor gene Drg-1 down-regulates the expression of activating transcription factor 3 in prostate cancer. Cancer Res.

[31] Kovacevic Z, Menezes SV, Sahni S, Kalinowski DS, Bae DH, Lane DJ, Richardson DR (2016). The metastasis suppressor, N-MYC downstream-regulated Gene-1 (NDRG1), down-regulates the ErbB family of receptors to inhibit downstream oncogenic signaling pathways. J Biol Chem.

[32] Lee JC, Chung LC, Chen YJ, Feng TH, Juang HH (2014). N-myc downstream-regulated gene 1 downregulates cell proliferation, invasiveness, and tumorigenesis in human oral squamous cell carcinoma. Cancer Lett.

[33] Sun J, Zhang D, Bae DH, Sahni S, Jansson P, Zheng Y, Zhao Q, Yue F, Zheng M, Kovacevic Z, Richardson DR (2013). Metastasis suppressor, NDRG1, mediates its activity through signaling pathways and molecular motors. Carcinogenesis.

[34] Schmidt EM, Gu S, Anagnostopoulou V, Alevizopoulos K, Foller M, Lang F, Stournaras C (2012). Serum- and glucocorticoid-dependent kinase-1-induced cell migration is dependent on vinculin and regulated by the membrane androgen receptor. FEBS J.

[35] Ben-Sahra I, Howell JJ, Asara JM, Manning BD (2013). Stimulation of de novo pyrimidine synthesis by growth signaling through mTOR and S6K1. Science.

[36] Herdy B, Jaramillo M, Svitkin YV, Rosenfeld AB, Kobayashi M, Walsh D, Alain T, Sean P, Robichaud N, Topisirovic I, Furic L, Dowling RJ, Sylvestre A (2012). Translational control of the activation of transcription factor NF-kappaB and production of type I interferon by phosphorylation of the translation factor eIF4E. Nat Immunol.

[37] Melotte V, Qu X, Ongenaert M, van Criekinge W, de Bruine AP, Baldwin HS, van Engeland M (2010). The N-myc downstream regulated gene (NDRG) family: diverse functions, multiple applications. FASEB J.

[38] Sommer EM, Dry H, Cross D, Guichard S, Davies BR, Alessi DR (2013). Elevated SGK1 predicts resistance of breast cancer cells to Akt inhibitors. Biochem J.

[39] Garcia-Martinez JM, Alessi DR (2008). mTOR complex 2 (mTORC2) controls hydrophobic motif phosphorylation and activation of serum- and glucocorticoid-induced protein kinase 1 (SGK1). Biochem J.

[40] Spitz DR, Sim JE, Ridnour LA, Galoforo SS, Lee YJ (2000). Glucose deprivation-induced oxidative stress in human tumor cells. A fundamental defect in metabolism?. Ann N Y Acad Sci.

[41] Leslie NR, Bennett D, Lindsay YE, Stewart H, Gray A, Downes CP (2003). Redox regulation of PI 3-kinase signalling via inactivation of PTEN. EMBO J.

[42] Lang F, Bohmer C, Palmada M, Seebohm G, Strutz-Seebohm N, Vallon V (2006). (Patho)physiological significance of the serum- and glucocorticoid-inducible kinase isoforms. Physiol Rev.

[43] Santag S, Siegel F, Wengner AM, Lange C, Bomer U, Eis K, Puhler F, Lienau P, Bergemann L, Michels M, von Nussbaum F, Mumberg D, Petersen K (2017). BAY 1143269, a novel MNK1 inhibitor, targets oncogenic protein expression and shows potent anti-tumor activity. Cancer Lett.

[44] Beggs JE, Tian S, Jones GG, Xie J, Iadevaia V, Jenei V, Thomas G, Proud CG (2015). The MAP kinase-interacting kinases regulate cell migration, vimentin expression and eIF4E/CYFIP1 binding. Biochem J.

[45] Waskiewicz AJ, Flynn A, Proud CG, Cooper JA (1997). Mitogen-activated protein kinases activate the serine/threonine kinases Mnk1 and Mnk2. EMBO J.

[46] Oh YM, Park HB, Shin JH, Lee JE, Park HY, Kho DH, Lee JS, Choi H, Okuda T, Kokame K, Miyata T, Kim IH, Lee SH (2015). Ndrg1 is a T-cell clonal anergy factor negatively regulated by CD28 costimulation and interleukin-2. Nat Commun.

