# Supplementary material for: Oncogenic MNK signalling regulates the metastasis suppressor NDRG1
Source: Oncotarget. 2017 May 2;8(28):46121–35. doi: 10.18632/oncotarget.17555 (PMC5542254; doi:10.18632/oncotarget.17555)
Supplement: Supplementary file 1 [file oncotarget-08-46121-s001.pdf]

Oncogenic MNK signalling regulates the metastasis suppressor NDRG1

SUPPLEMENTARY FIGURE

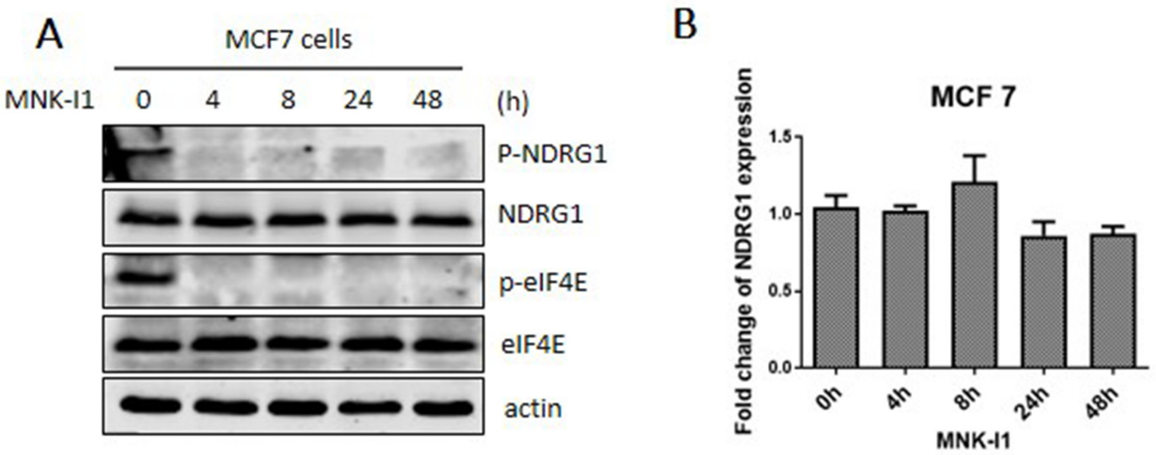

Supplementary Figure 1: MNK inhibition blocks NDRG1 phosphorylation but not its expression in MCF7 cells. MCF7 cells were treated with 5 µM MNK-I1 for 4, 8, 24 or 48 h. Protein levels **(A)** were analysed by western blot with the indicated antibodies, and levels of NDRG1 mRNA **(B)** by RT-qPCR.
